# The gap between bachelor’s degree graduates in health informatics and employer needs in Saudi Arabia

**DOI:** 10.1186/s12909-023-04442-7

**Published:** 2023-06-26

**Authors:** Haitham Alzghaibi

**Affiliations:** Department of Health Informatics, College of Public Health and Health Informatics, Qassim University, Albukayriah, 52741 Saudi Arabia

**Keywords:** Health informatics, Bachelor’s degrees, Career pathway, Employment, Education

## Abstract

**Background:**

In the field of health informatics (HI), there is a crucial gap between employers’ needs and the output of academic programmes. Although industrial organisations and government agencies recognise the importance of training and education in the development and operation of health-information systems, advancements in educational programmes have been comparatively slow in terms of investment in healthcare information technology. This study aims to determine the gap between employer demands and academic programmes in HI in Saudi Arabia.

**Methods:**

This mixed-methods study collected both qualitative and quantitative data. A qualitative content analysis was performed to identify the role of advertised HI jobs using two sources: Google and LinkedIn. In addition, university websites were searched to determine job opportunities for graduates with a bachelor’s degree in HI. Next, a quantitative, cross-sectional self-report questionnaire was administered to validate the findings of the qualitative data. Data obtained were analysed using SPSS, N-Vivo, and Microsoft Excel.

**Results:**

The study’s data were obtained from four sources: Google search engine, LinkedIn, five Saudi university websites, and 127 HI experts. The results show a discrepancy between academic programmes’ outputs and employer recruitment needs. In addition, the results reveal a preference for post-graduate degrees, either a master’s or PhD degree, with a bachelor’s degree in a health or medical discipline.

**Conclusions:**

Employers tend to prefer applicants with a bachelor’s degree in computer science or information technology over those with a degree in HI. Academic programmes should incorporate more practical applications and provide students with a thorough understanding of the healthcare industry to better equip them as efficient future HI professionals.

## Background

Health informatics (HI) is an invaluable field of study in the modern age as it combines the medical industry, data management, and technology, and enables practitioners to make decisions that best serve their patients. Furthermore, HI is vital in the early detection and management of chronic diseases [1]. It helps healthcare providers manage patient data, identify high-risk patients, and develop interventions that target the specific needs of patients. It also allows healthcare professionals to share patient data and collaborate on care plans, which leads to more coordinated and effective care [2]. HI plays a crucial role in healthcare management and administration. It helps healthcare providers manage resources efficiently, track inventory, and monitor key performance indicators for quality assurance [3]. It also ensures the integration of clinical and administrative data, leading to better decision-making and resource allocation [3]. Moreover, HI ensures that patient information is secure and protected. Privacy is a top priority in healthcare, and HI provides a secure platform for healthcare providers to manage patient data and ensures that the data are only accessible to authorized personnel [2, 3].

Within the evolving technology and the healthcare landscape, the skills and knowledge to stay ahead of these changes are becoming increasingly important [1, 4]. Unfortunately, a growing gap exists between employers’ needs and HI students’ knowledge at graduation [4, 5]. Owing to the rising demand for HI services, employers must find professionals with the required skills and expertise in the job market [6]. Although IT professionals who have been trained or have experience in HI bring valuable skills and experience to the table, employers still report difficulty finding suitable candidates. Students preparing to enter the workforce lack an understanding of healthcare needs and the skills to solve problems specific to the healthcare industry [7]. This inadequacy points to a misalignment between the current educational curriculum and employer requirements [5].

HI education lacks adequate on-the-job training to prepare graduates for industry roles. Despite students learning additional skills and developing those already learned in school, the acquired skills are often not fundamental to their job [8–10]. Employers want to hire candidates who can function efficiently in the job environment and are up-to-date with the latest tools and technologies [8]. They need graduates who demonstrate a hands-on approach and possess a certain level of practical skills in addition to their theoretical knowledge [7]. The increasing demand for people with an understanding of HI induces a growing concern among employers owing to the scarcity of highly skilled professionals.

With constantly changing technology, employers need employees to keep abreast with advancements in this field [5, 10]. This issue can be addressed by increasing the number of practical applications and implementing HI activities in the curriculum. Furthermore, students should be exposed to the healthcare industry outside the classroom to increase their exposure to real-world settings [7, 9].

### The Saudi Commission for Health Specialists as a supervisory and regulatory organisation

HI is an increasingly important field in the healthcare industry because it stores and provides vital access to patient information, medical records, and other important medical data. To ensure that HI graduates have the necessary qualifications to maintain and protect patient data, the Saudi Commission for Health Specialists (SCFHS) has developed a classification process to prepare graduates for their roles in the healthcare industry [5, 8–10]. This process involves both theoretical and practical skills, such as examining students’ knowledge in relevant fields (e.g., healthcare information technology [IT], databases, and computer programming) and completing a project that demonstrates students’ skills and competencies in HI [8].

The students who participate in these SCFHS projects research and develop HI applications or programmes. These applications exemplify HI skills such as the use of databases, programming, security, and other relevant fields. The projects are evaluated by a committee of HI experts and serve as a key factor in the committee’s decision to certify the students as qualified for their role in the health industry. Once the students are deemed qualified, they receive a certificate of specialisation in HI from the SCFHS that is officially recognised and makes them eligible to apply for work in the health industry. This certification is a valuable addition to students’ resumes, and allows employers to easily identify a student’s qualifications and expertise in HI [9, 11].

### The gap between employers’ needs and academic programmes in HI and the role of professional bodies

A critical issue in the field of HI is the gap between employers’ needs and the output of academic programmes. Despite industrial organisations and government agencies recognising the importance of training and education in developing and operating health information systems, progress has been comparatively slow in terms of investment in healthcare information technology [5, 7]. In the early 1990s, countries had very few established HI programmes because of the slow response of higher education institutions related to the information revolution in healthcare [12]. The continuous need to provide education and training for the workforce in healthcare information systems and information management is one of the reasons for the delayed progress in this area. Individual staff members require different levels of training and education. Therefore, the differing skills and abilities of employees and their aspirations for further training, education, and qualifications need to be considered [7].

Globally, employers expect students from different countries to have the same basic knowledge and skills in healthcare information and technology [7]. However, the interdisciplinary nature of HI generates diverse perspectives on course content, admission standards, and requirements for degree programmes. For example, 30% of the medical and HI programmes in the United States are interdepartmental, which means that these academic programmes could face administrative barriers [13]. Additionally, the diversity of healthcare information provides a wide spectrum of topics across various HI programmes. Programmes’ emphasis also vary by country. For example, in Germany, a greater focus is placed on clinical issues reflecting the tradition of medical informatics. However, in the United States, there is a stronger emphasis on healthcare administration and health policies [12].

Owing to the accelerating demand for HI professionals and the increasing availability of a range of HI programmes that provide graduates with different types of expertise [14], maintaining high standards and quality education in this field is important. National accreditation committees generally evaluate HI educational programmes. For instance, in the United States, the Commission on Accreditation for HI and Information Management Education (CAHIIM) establishes and enforces quality accreditation standards for HI and health information management programmes (CAHIIM, 2017) with the support of the American Medical Informatics Association (AMIA).

Organisations like the International Medical Informatics Association (IMIA) are responsible for making recommendations on biomedical and HI education to guide curriculum development [14, 15]. The IMIA has international expertise in the field of education and helps educational institutions define the content of their curricula and the knowledge and skills necessary for different categories of HI specialists [14, 15]. The organisation has also established an accreditation service for international HI education benchmarks. The IMIA accreditation is provided in addition to, and not as a substitute for, accreditations available in other countries [15]. This accreditation allows educational institutions to attain international status and become more competitive and attractive to students [16]. It gives students mobility so that they can study in another country and return after completing their studies overseas, or work internationally [16].

Professional bodies, such as the AMIA, support HI practitioners by sharing research and best practices and advancing HI as a profession [15]. The AMIA has developed a professional code of ethics and a top-ranking scientific research journal, the Journal of the American Medical Informatics Association, that publishes articles on the growing HI workforce and the needs of specialty areas [17]. Similar to AMIA, the Saudi Association for HI (SAHI) supports HI practitioners in Saudi Arabia (SA) [8].

SA’s healthcare sector has undergone a major transformation in recent years, with a focus on improving the quality of healthcare services. Consequently, HI has gained importance as a means to improve the delivery of healthcare services [5]. The field is experiencing rapid growth, with a large number of academic programmes in HI being offered at various universities. However, there is still a gap between the output of academic programmes in HI and the healthcare sector’s employment needs. The gap is caused by various factors, such as the lack of industry collaboration in curriculum development, limited job opportunities, and inadequate training and development programmes.

This study aims to determine the gap between employer demand and academic programmes in HI in SA. This gap must be addressed to ensure that students are better equipped to enter the industry. Incorporating increased practical applications and providing students with a thorough understanding of the healthcare industry can give them the resources needed to become efficient future HI professionals.

## Methods

The current study employs a convergent parallel mixed-methods design [18]. First, a qualitative content analysis [19] was performed to identify the advertised HI roles using two sources: Google and LinkedIn. In addition, university websites were searched to determine job opportunities for graduates with a bachelor’s degree in HI. Second, a quantitative, cross-sectional [20], self-report questionnaire was administered to HI experts, academics and researchers to compare the findings of the qualitative data.

A mixed methods approach was selected owing to its ability to generate both quantitative and qualitative data. The combination of quantitative and qualitative data yields richer and more comprehensive data, which, in turn, helps the researcher gain a deeper understanding of the research problem and provides more nuanced insights. Additionally, a mixed methods design provides greater validity as it triangulates the results of the different research methods, thus offering more reliable results. However, a mixed methods design requires a significant investment of resources, both in terms of time and effort. It is more time-consuming to conduct both quantitative and qualitative research; thus, it requires more funds to hire additional researchers, transcribers, and data analysts.

### Population and sampling

#### Qualitative

In January 2023, a comprehensive search was performed using two popular tools, Google and LinkedIn, to identify jobs advertised for HI specialists. Several inclusion and exclusion criteria were adopted to obtain accurate results (Table 1).

**Table 1:** Inclusion and exclusion criteria for job title selection

Included	Excluded
Health informatics	Medical informatics
Bachelor’s degree	Health information management
Full-time jobs	Clinical informatics
Job listed in the last 10 years	Master’s in health informatics
Government jobs	PhD in health informatics
Private jobs	Part-time jobs
	Job listed before 2013
	Volunteer jobs
	Short-term contracts (three months or less)

Furthermore, five universities were selected to determine the job opportunities designed for graduates with a bachelor’s degree in HI. These universities were selected according to the exclusion and inclusion criteria outlined in Table 2.

**Table 2:** Inclusion and exclusion criteria for university selection

Included	Excluded
University that provides a bachelor’s degree in HI	University that has insufficient information online
University that has sufficient information online	University that only provides a master’s degree in HI
University that retains records of alumni for at least three years	University that only provides a PhD degree in HI
Private university	Newly established programmes
Government university	Bachelor’s degree programmes that have no alumni yet

#### Quantitative

The target population included all HI experts in SA (n = 1029). The study population was determined based on two WhatsApp groups and one Telegram group. These collaborative groups are officially used by the SAHI and the Saudi Ministry of Health. As this study was based on a state-wide population, convenience sampling was conducted based on an online questionnaire [21]. Convenience sampling is an effective approach when accessing the entire target population is difficult [21]. In addition, it assists in minimising the costs and time associated with state-wide studies [21]; however, despite being considered easy to conduct and efficient regarding cost and time, it can lead to selection bias [22]. Consequently, samples from different categories, such as healthcare professionals, non-healthcare professionals, and academics, were selected. The questionnaire was distributed to 280 participants based on the sample size calculation, with a 95% confidence level and a 5% margin of error.

### Data collection tools and procedures

#### Qualitative data collection

**Fig. 1:**
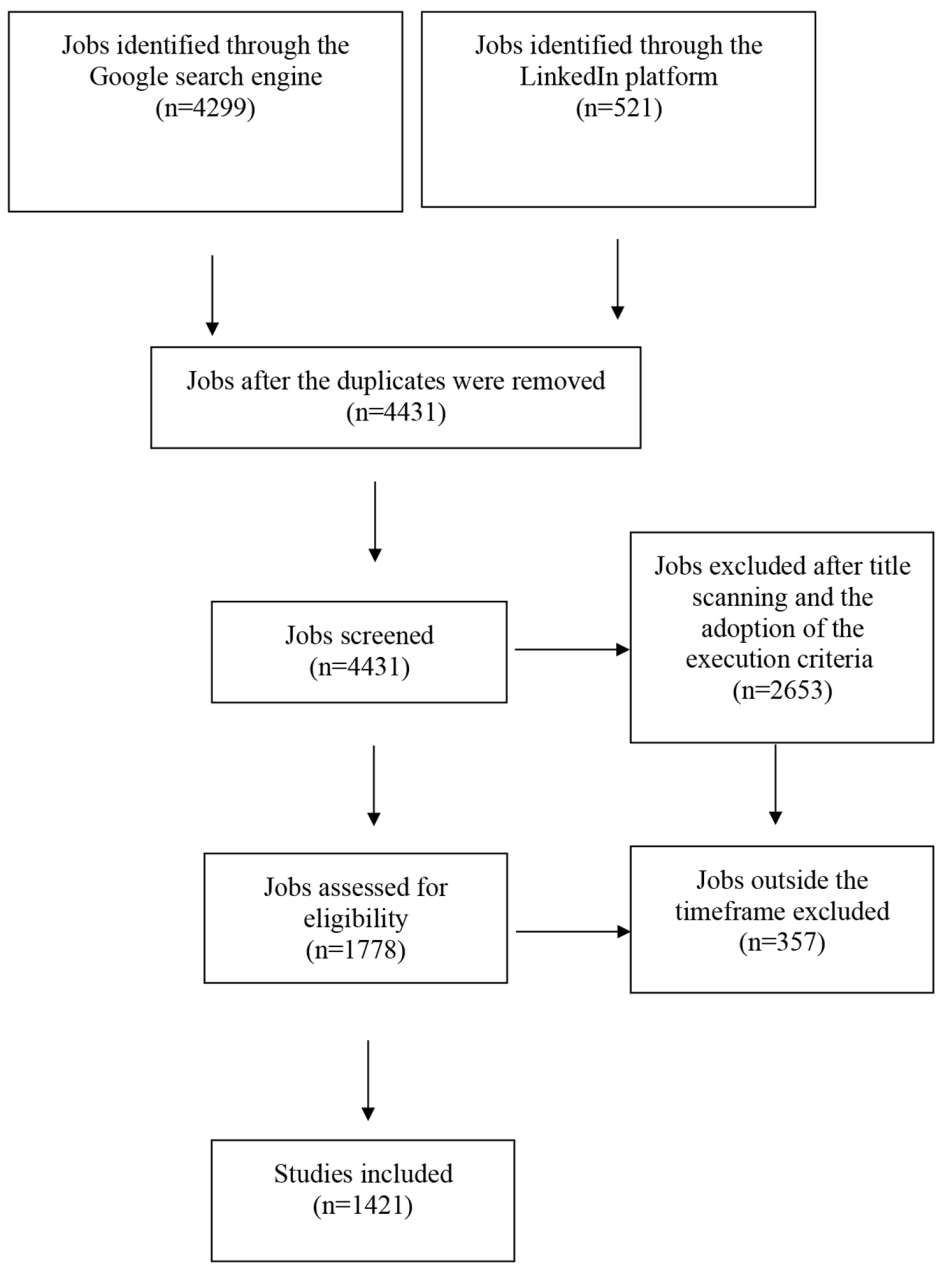
Inclusion of jobs

At this stage, the comprehensive search included the following keywords: ‘health informatics’, ‘health informatics specialists’, ‘bachelor’s in health informatics’, ‘health informatics job’, ‘looking for health informatics’, ‘people holding health informatics’, and ‘experience in health informatics’. As presented in Figs. 1 and 1421 job titles were included in the analysis after associating the exclusion criteria with the above keywords and performing searches twice in English and once in Arabic.

#### Quantitative data collection

Data collection was performed from 20 to 2023 to 25 February 2023. A questionnaire was designed and distributed online using Google Forms. Google Forms was selected as a platform because it is free, easy to use, and accessible for both researchers and participants. The designed questionnaire was divided into three sections and comprised seven questions: three demographic questions and four questions related to the HI profession. The average time to complete the questionnaire was approximately 10–15 min. The first section included a letter explaining the nature and aims of the study, the importance of participation, ways to participate, and assurance that the responses would be anonymous. This section also included a statement informing participants of their right to withdraw at any time without clarification. Consent was required to proceed to the next section of the questionnaire. The second section included three questions on demographic information, such as gender, position, and level of education. The last section comprised four questions, one requiring a ‘yes’ or ‘no’ response and the remaining requiring multiple responses. All the multiple responses included in the last three questions were extracted from the qualitative data, and participants were allowed to select more than one response to validate the findings of qualitative data.

Although the questionnaire items were added based an analysis of the qualitative data, a pilot study was conducted by sending the questionnaire to a small number of the targeted population (n = 6) to ensure that the questionnaire was readable, comprehensive, clear, reliable, and appropriate for the study aim. The preliminary questionnaire comprised five questions.

1. Did you understand the questionnaire?
2. Were there any items that you did not understand and why?
3. Are there any items you think should be included or excluded?
4. Would you like to make any other comments about the questionnaire?

None of the participants in the pilot study suggested any inclusions or exclusions to the questionnaire. Overall, the preliminary pilot questionnaire was understandable and easy to complete. The pre-test was conducted for two weeks, from 12 to 2023 to 25 January 2023.

### Data analysis

#### Qualitative

Although a content analysis mainly produces qualitative data based on text [19], this study used Microsoft Excel (ME) to analyse the obtained data [23] and calculate the number and percentage of job title appearances [23]. ME is also fully compatible with both Arabic and English and can deal with short texts, such as job titles or job opportunities.

#### Quantitative

IBM SPSS Statistics 29 was used to analyse the quantitative data [24]. All the data in the data collection instrument were numerically coded using SPSS. Cronbach’s alpha test was used to examine the questionnaire’s reliability. A descriptive analysis was performed on the entire dataset to analyse multiple responses [24]. The most frequently selected answers were categorised into groups and then displayed in tables with the most frequent answers appearing together, followed by less popular answers. The multiple responses were also split into individual terms to determine the most frequently selected terms.

Frequency tables were used to express the results, and t-tests were performed to determine whether there was a significant difference between the responses of the male and female respondents. ANOVA was performed for a group of questions to examine the differences between various education and occupation groups. Chi-square tests and cross-tabulation tables were used to show which groups had different answers. A p-value of less than 0.05 was considered statistically significant [24].

## Results

Of the 280 HI experts, 127 (45%) completed the online questionnaire (Table 3). Table 3 summarises the analysis of the respondent’s demographic characteristics, including the distribution of the respondents’ occupations, gender, and education levels. Of the total who responded to the questionnaire, 65.14% of respondents were healthcare professionals, 26.44% were academics, 3.94% were IT professionals, and 3.15% were researchers. The majority of the respondents were men (65.14%). Regarding respondents’ education level, 57.41% had a bachelor’s degree, 31.89% had a master’s degree, and only 9.4% had a PhD (Table 3).

**Table 3:** Participants’ responses to demographic questions

Occupation	Frequency	Percentage
Academic	34	26.4
Healthcare professional	84	65.1
IT professional	5	3.9
Researcher	4	3.1
Total	127	100.0
**Gender**	**Frequency**	**Percentage**
Female	43	33.3
Male	84	65.1
Total	127	100.0
**Level of education**	**Frequency**	**Percentage**
Bachelor	74	57.4
Master	41	31.8
PhD	12	9.3
Total	127	100.0

**Fig. 2:**
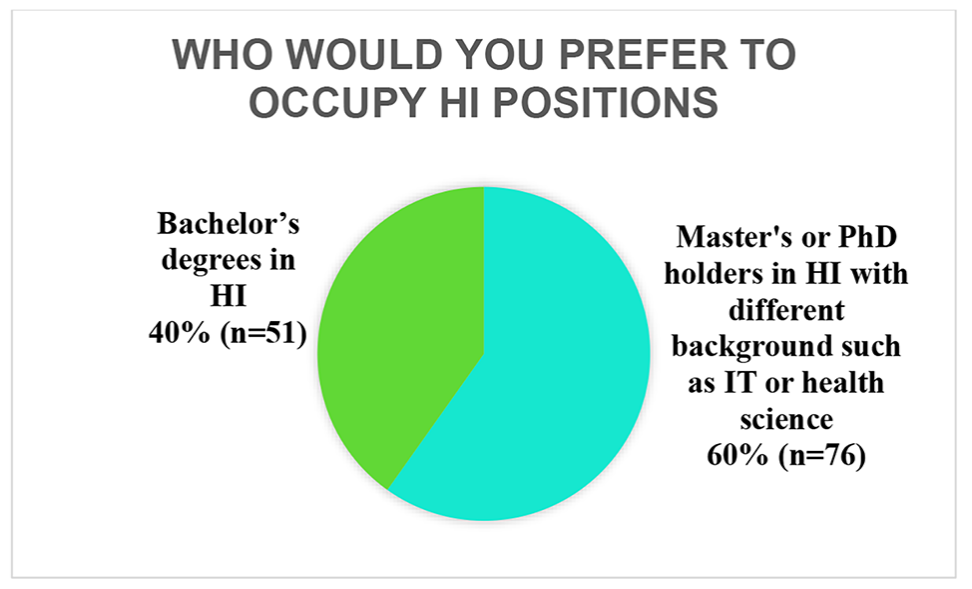
Preferred HI positions

As seen in Fig. 2, approximately 60% of the respondents reported that they would hire applicants with a master’s or PhD degree in HI. Less than half of the participants said they would hire applicants with a bachelor’s degree in HI.

Regarding the most appropriate disciplines for a graduate degree, 10.2% of the respondents answered medicine, nursing, health science and public health, medical science, IT, and computer science; 9.4% answered health science and public health, management, IT, and computer science; 8.7% answered medicine, nursing, health science and public health, medical science, management, engineering, IT, and computer science; 7.9% chose only health science and public health; and 7.9% answered health science and public health, IT, and computer science. As can be seen in Table 4, the most frequent response was IT (100), followed by computer science (96), and health science and public health (89). Healthcare science and management and medical science were both chosen by 62 respondents. Nursing appeared 47 times in the responses.

**Table 4:** Most appropriate discipline for HI

Description based on multiple responses	Percentage	Discipline based on a single response	Number of appearances	Percentage
Medicine, nursing, health science and public health, medical science, IT and computer science	10.2%	Health science and public health	89	19%
Health science and public health, management, IT and computer science	9.4%	Management	62	14%
Medicine, nursing, health science and public health, medical science, management, engineering, IT and computer science	8.7%	Computer science	96	21%
Health science and public health	7.9%	IT	100	22%
Health science and public health, IT and computer science	7.9%	Medical science	62	14%
Other	55.9%	Nursing	47	10%
	100%	Total	456	100%

Regarding the question on which of the preferred occupations are appropriate for HI practitioners, 31.5% of the respondents answered physicians, nurses, pharmacists, lab technicians, and administration and management science; 11.8% of the respondents answered computer science and IT; 9.4% answered physicians, nurses, pharmacists, lab technicians, computer science, and IT; 8.7% of the respondents answered physicians, nurses, administration and management science, computer science, and IT; and 6.3% answered nurses, computer science, and IT. It is also useful to examine the single responses. Table 5 shows that the most frequently given answer was computer science engineer (103 respondents), followed by IT (100 respondents), nurses (86), and physicians (73). Only three respondents selected health information management.

**Table 5:** Occupations appropriate to be HI practitioners

Occupation based on multiple responses	Percentage	Occupation based on a single response	Number of appearances	Percentage
Physicians, nurses, pharmacists, lab technicians, administration and management science, radiologists, project managers, computer science and IT	31.5%	Administration and management	58	10%
Computer science and IT	11.5%	Computer science engineer	103	18%
Physicians, nurses, pharmacists, lab technicians, computer science and IT	9.4%	IT specialist	100	18%
Physicians, nurses, radiologists, administrative, computer science and IT	8.7%	Health information manager	3	1%
Nurses, computer science and IT	6.3%	Physicians	73	13%
Other	32.6%	Nurses	86	15%
Total	100%	Project Manager	20	4%
		Pharmacists	59	10%
		Radiologists	63	11%
		Total	565	100%

Table 6 shows that 17 HI roles emerged after analysing multiple resources. The survey’s findings revealed that health data scientist (8%), telemedicine operational specialist (8%), mHealth and web applications specialist (8%), Health information system (HIS) developer (8%), health information manager (8%), and academic (8%) were the top demanded roles for HI graduates. Employers and employment platforms websites showed that health data scientist (11%), HIS analyst (10%), data analytics (10%), health information manager (18%), and academic (13%) were the most demanded roles for graduates with a bachelor’s degree in HI. Table 6 also shows that 10% of medical coders, HIS developers, health information managers, and researchers agreed on all selected government universities as job opportunities for HI graduates.

**Table 6:** Multiple perspectives of HI roles

	HI Role	Appearance in our study	Percentage based on multiple responses	Percentage based on a single response	Appearance in the Saudi market	Percentage	Appearance in Saudi universities (n = 5)	Percentage
1.	Administrative	45	35%	3%	31	8%	2	4%
2.	Data entry and receptionist	29	23%	2%	3	1%	0	0%
3.	Health data scientist	108	85%	8%	44	11%	1	2%
4.	Telemedicine operation specialist	110	87%	8%	13	3%	0	0%
5.	mHealth and web applications specialist	109	86%	8%	6	1%	4	8%
6.	HIS analyst	78	61%	5%	42	10%	4	8%
7.	Medical coder	75	59%	5%	11	3%	5	10%
8.	Data analytics	102	80%	7%	41	10%	5	10%
9.	IT Project manager	90	71%	6%	29	7%	4	8%
10.	HIS developer	114	90%	8%	21	5%	5	10%
11.	Technical support in healthcare organisation	71	56%	5%	22	5%	1	2%
12.	Health information manager	118	93%	8%	72	18%	5	10%
13.	Datacentre manager	69	54%	5%	25	6%	0	0%
14.	Academic	113	89%	8%	55	13%	2	4%
15.	Researcher	104	82%	7%	12	3%	5	10%
16.	Quality assurance specialists in healthcare	34	27%	2%	2	1%	4	8%
17.	Insurance and reimbursements	52	41%	4%	7	2%	4	8%
Total		1421	1119%	100%	405	100%	51	100%

## Discussion

This study aimed to determine the gap between academic outcomes and employer expectations and needs in SA. A content analysis was initially performed to identify the most frequently advertised role for HI over the last five years. The identified list was compared with job opportunities on selected government university websites. The comparison process illustrated the variance in priorities between market demand and university goals. For instance, while the most demanded HI occupation in the Saudi market is an academic position, only two of the five universities thought their graduates could occupy these positions. Among the 44 advertised jobs seeking health data scientists who had graduated with a bachelor’s degree in HI, only one university believed that its graduates could occupy these positions. Owing this variance, consolidating bachelor’s degree programmes in HI is strongly recommended to 8 job opportunities for the graduates and reduce the gap between graduates’ attributes and employers’ demands. Programme directors and curriculums designers may need to benchmark the proposed HI skill competencies framework developed by Almalki, Jamal [9] to reduce the identified gap. Almalki, Jamal [9] suggested that the competencies framework should focuses on five main skills: leadership and management, information communication technology, education and research, health data analysis, and health science. Although, the majority of determined job opportunities can be linked with the HI skill competencies framework, some skill requirements are not adequately met by the Saudi universities.

However, the Saudi market and government universities agree that roles such as HIS developers, medical coders, and health data analytics are the most appropriate for HI graduates. Notably, although several advertised jobs for HI graduates employed data centre managers, data entry and receptionists, and telemedicine operation specialists, government universities assumed that their graduates were unsuitable for these roles.

For further validation, 127 HI experts were surveyed about the most suitable HI jobs for graduates with a bachelor’s degree. The results of the analysis (Table 4) illustrate a high level of agreement with the demands of the Saudi market. Participants’ demographic distribution, which shows that most participants were healthcare professionals, justifies that they may belong to organisations in the analysed Saudi markets. The participants were also asked to give their opinion on the most appropriate qualification for obtaining an HI job. Surprisingly, more than half of the participants thought a master’s or PhD degree in HI with pre-qualifications in other health-related disciplines would be more desirable than a bachelor’s degree in HI. The participants were also asked about the most common combinations of disciplines with HI. The results showed that HI graduates with pre-qualifications in health science, computer science, and IT were considered preferable for HI jobs. However, the inferential test on their responses to this question revealed no significant differences between the groups. In the same context, we asked participants about professions that fit HI jobs. The findings show that computer science engineers and IT specialists are in greater demand than other professionals. Our findings also revealed a moderate demand for nurses, physicians, and radiologists. These results reflect the Saudi market’s outlook for hiring HI specialists, which exhibits a preference for computer engineers and information technology specialists over HI graduates. These findings raise concerns about the future of HI graduates, especially those with a bachelor’s degree in HI, and their ability to obtain appropriate jobs that match their qualifications. This may increase the gap between academic programmes and employers.

The current study findings are consistent with those of Zakaria, Zakaria [5] who identified a considerable gap between academic outcomes and employer needs. In addition, both studies revealed a mismatch between advertised HI jobs and actual employment, despite the attempts by SCFHS and SAHI to classify HI graduates, make them eligible for roles in the HI industry, and develop career pathways for them [5, 8–10]. Although the findings of this study reveal that employers prefer employees with a master’s or PhD degree in HI with pre-qualifications in other health-related disciplines, previous studies have illustrated that academic programmes in HI should consider different classifications, such as nursing informatics, clinical informatics, medical informatics, and biomedical informatics [9, 15]. Therefore, future studies should compare bachelor graduates in HI with other classifications. Researchers should also consider qualitative approaches via semi-structured interviews or focus groups with selected informants drawn from the same population to seek further clarification and justifications that could assist in reducing the identified gap. Moreover, future researchers may need to conduct further investigations to explore tutors, programs’ administrators, and curriculum designers’ awareness about the gap between academic programs and employers’ needs.

In this study, the gap was determined based on the triangulation of data sources, such as a search engine and an employment platform, university websites, and expert surveys, which improved the validity of the findings. Although a few studies have identified the gap between employer needs and academic programmes, this study is the first to focus on graduates with a bachelor’s degree in HI.

This study has a few limitations. First, it was difficult to calculate the total population of HI experts as there is no database for them. Next, the role of HI was determined using one search engine (Google) and one employment platform (LinkedIn). Finally, although most participants were from healthcare organisations that advertised HI jobs, it was difficult to identify those who were directly involved in the employment process.

## Conclusions

This study identified the gap between employer demands and academic programme outcomes. Although numerous universities provide a bachelor’s degree in HI, employers tend to employ applicants with a master’s or PhD in HI and a graduate degree in a health or medical discipline. Graduates with a bachelor’s degree in computer science or IT are more likely to occupy HI jobs. Finally, Saudi universities have failed to address the gap between academic programmes and employer expectations in the Saudi market because some of the identified job titles, such as telemedicine operation specialists, data entry and receptionists, or data centre managers, were not included as job opportunities for graduates with a bachelor’s degree in HI.

## Data Availability

The datasets used and analysed in the current study are available from the corresponding author upon reasonable request.
